# Microarray comparative genomic hybridization detection of chromosomal imbalances in uterine cervix carcinoma

**DOI:** 10.1186/1471-2407-5-77

**Published:** 2005-07-09

**Authors:** Alfredo Hidalgo, Michael Baudis, Iver Petersen, Hugo Arreola, Patricia Piña, Guelaguetza Vázquez-Ortiz, Dulce Hernández, José González, Minerva Lazos, Ricardo López, Carlos Pérez, José García, Karla Vázquez, Brenda Alatorre, Mauricio Salcedo

**Affiliations:** 1 Laboratorio de Oncología Genómica, Unidad de Investigación Médica en Enfermedades Oncológicas, Centro Médico Nacional Siglo XXI-IMSS, México; 2 Division of Pediatric Haematology/Oncology, University of Florida, Gainesville, USA; 3 Institute of Pathology, University Hospital Charité, Berlin, Germany; 4 Servicio de Epidemiología, Hospital de Oncologia, Centro Médico Nacional Siglo XXI-IMSS, México; 5 Clínica de Displasias, Hospital de Gineco-Obstetrica No. 4, Luis Castelazo Ayala-IMSS, México; 6 Departamento de Patología, Facultad de Medicina UNAM-Hospital General de México, SS, México; 7 Laboratorio de Biología Teórica, Departamento de Investigación, Universidad La Salle, México; 8 Instituto Nacional de Medicina Genomica, Secretaria de Salud, Mexico

## Abstract

**Background:**

Chromosomal Comparative Genomic Hybridization (CGH) has been applied to all stages of cervical carcinoma progression, defining a specific pattern of chromosomal imbalances in this tumor. However, given its limited spatial resolution, chromosomal CGH has offered only general information regarding the possible genetic targets of DNA copy number changes.

**Methods:**

In order to further define specific DNA copy number changes in cervical cancer, we analyzed 20 cervical samples (3 pre-malignant lesions, 10 invasive tumors, and 7 cell lines), using the GenoSensor microarray CGH system to define particular genetic targets that suffer copy number changes.

**Results:**

The most common DNA gains detected by array CGH in the invasive samples were located at the *RBP1-RBP2 *(3q21-q22) genes, the sub-telomeric clone C84C11/T3 (5ptel), D5S23 (5p15.2) and the *DAB2 *gene (5p13) in 58.8% of the samples. The most common losses were found at the *FHIT *gene (3p14.2) in 47% of the samples, followed by deletions at D8S504 (8p23.3), *CTDP1-SHGC*- 145820 (18qtel), *KIT *(4q11-q12), D1S427-*FAF1 *(1p32.3), D9S325 (9qtel), *EIF4E *(eukaryotic translation initiation factor 4E, 4q24), *RB1 *(13q14), and DXS7132 (Xq12) present in 5/17 (29.4%) of the samples.

**Conclusion:**

Our results confirm the presence of a specific pattern of chromosomal imbalances in cervical carcinoma and define specific targets that are suffering DNA copy number changes in this neoplasm.

## Background

Uterine cervix carcinoma (UCC) represents the second cause of death among the female population worldwide. The fact that more than 99% of all the cervical invasive tumors are positive for infection with high risk human papillomavirus (HPV) suggests that this is one of the most important factors for the development of this neoplasm [1,2]. These viruses can induce cellular transformation by several mechanisms; the viral oncoproteins E6 and E7 can interact with cellular proteins involved in important cellular functions, such as tumor suppression, apoptosis, cell cycle control, genomic instability, transcriptional regulation and immune evasion [3].

The induction of genomic instability by HPV seems to be particularly important for the establishment and development of an invasive tumor [4,5] since this increased genomic plasticity would generate cellular clones with enhanced transforming and invasive potential [6].

Metaphase comparative genomic hybridization (mCGH) has been applied to study different stages of this tumor [4,7-19], detecting specific patterns of chromosomal imbalances that arises very early during the development of cervical carcinoma, suggesting that the gain of chromosome 3q is one of the most important genetic alteration that defines the transition from a pre-malignant lesion to an invasive carcinoma [4]. Some of these imbalances have been related to specific clinical behaviors, such as the presence of lymph node metastases [9]. However, given the spatial resolution of mCGH [20], little is known about the identity of specific genes that might be the targets of regional chromosomal imbalances. Matrix-based CGH or array CGH overcomes this problem increasing the sensitivity for the detection of DNA copy number changes at specific loci, through the use of well defined genomic DNA fragments whose mapping location is known, arrayed onto a solid surface [21-23], thereby achieving a resolution of copy number imbalances up to the single gene level.

In order to refine the patterns of chromosomal imbalances present in cervical carcinoma, and trying to identify specific genes that might be targets of copy number changes in this tumor, we applied microarray CGH on 20 uterine cervix-derived samples (three pre-malignant lesions, 10 invasive tumors and seven UCC derived cell lines) to detect DNA copy number changes at the single gene level.

## Methods

### Cervical tissues

All described procedures have been evaluated and approved by the local committee of ethics of the Mexican Institute of Social Security (IMSS), and all samples were taken after informed consent from the patients. The pre-malignant lesions and the invasive tumors were collected by colposcopy-directed biopsies at the Gynecology Department of the Hospital General de México, Mexico City. The biopsies were divided in three sections. The central part was used for genomic DNA extraction using the Wizard Genomic kit (Promega, Madison, WI, USA), and the extremes were fixed with 70% ethanol overnight and paraffin embedded. Hematoxilin-eosin stained sections from these biopsies were analyzed in order to confirm the presence of at least 70% tumoral cells in the samples.

### Cell lines

The cell lines included in this study were: CasKi, SiHa, both positive for HPV16, and HeLa (HPV18) The CaLo and ViBo cell lines were established from stage IIB invasive tumors, while INBL and RoVa from a stage IVA tumor. These cells are HPV18 positive and were established from tumor explants at the laboratory of Cell differentiation and Cancer of the National University of Mexico [24]. The chromosomal CGH profiles of CaLo, ViBo, INBL and RoVa have been published recently [19].

### HPV detection and typing

HPV detection was carried out by PCR using the consensus primers MY09 and MY11 for the L1 region of the viral genome. After a 5 min. denaturation at 94°C, 100 ng of DNA were subjected to 35 amplification cycles with the following parameters: 94°C for 1 min., 55°C for 2 min. and 73°C for 3 min., with a final extension step of 7 min. at 72°C. The amplicon was labeled using the Big Dye sequencing kit and sequenced on an ABI371 sequencer (Applied Biosystems, Foster City, CA, USA). BLAST  sequence comparison was used in order to define the viral type.

### Microarray CGH

Microarray CGH was performed using the GenoSensor Array 300 system, following the manufacturer's instructions (ABBOT-Vysis, Downers Grove, IL, USA). Each array contains 861 spots, representing 287 chromosomal regions that are commonly altered in human cancer, such as telomeres, regions involved in microdeletions, oncogenes, and tumor suppressor genes. Briefly, 100 ng of genomic DNA were labeled by a random primer reaction during two hours. Tumor DNA was labeled with Cy3 and the normal female reference DNA with Cy5. After the labeling reaction, the probes were digested with DNAse at 15°C for one hr., followed by two ethanol-purifications; finally the probe size was checked by gel electrophoresis. The hybridization mixture consisted of 2.5 μl of each of the differentially labeled DNAs plus 25 μl of hybridization buffer provided in the kit. This mixture was denatured at 80°C for 10 min. at 80°C, followed by incubation at 37°C for one hr. Five μl of this probe were applied onto the spotted area of the array under a coverslip and hybridized in a humid chamber containing 50% formamide (FA)/2XSSC at 37°C for 72 hrs. After hybridization, the arrays were washed 3X in 50%FA/2XSSC at 40°C for 10 min/wash, followed by four 5 min. washes in 1XSSC at room temperature. Finally, the arrays were briefly rinsed in distilled water, mounted and counterstained in the dark for 45 min. with DAPI (4,6-diamino-2-phenylindole).

### Image capture and analysis

Array analysis was performed immediately after counterstaining using the GenoSensor scanner and software. This system generates a "genomic analysis report", indicating which chromosome regions in the array are involved in copy number changes, as well as a spreadsheet containing the data generated by a single experiment. In order to compare all the experiments, a database was created using the normalized, bias corrected, tumor/normal ratio value of each experiment [see additional file 1]. Since each spot in the array is present in triplicates, the median of the three spots of each probe in the array was calculated and its log2 transformed value was used for further analysis. A fluorescence ratio >1.25 (log2 = 0.32) was considered as a DNA gain, while DNA losses were scored when the ratio was <0.75 (log2 = -0.41). A ratio >2 (log2 = 1) was considered as a high copy number amplification.

## Results

### HPV detection and typing

One of the premalignant lesions was positive for HPV16 infection; one for HPV31 and the other for HPV58. In the invasive tumors, seven were positive for HPV16 and in three cases, we were not able to detect HPV sequences with the oligonucleotides we used for PCR amplification. As expected, CasKi and SiHa were positive for HPV16, while HeLa, INBL, CaLo, ViVo and RoVa were positive for HPV18.

### Microarray comparative genomic hybridization

All our samples, except one pre-malignant lesion, presented alterations, ranking from 1/287 (alterations/total targets in the array) in a pre-malignant lesion to 175/287alterations in the cell line RoVa. We found almost twice the number of DNA gains than DNA losses (571 vs. 298) and the average number of copy number alterations (ANCA=total number of alterations in the sample collective/total number of cases) was 43.45 per case.

One of the pre-malignant lesions did not show any alteration, while amplifications at *MSH2-KCNK12 *(2p22.3-2p22.1), *TCL1A *(14q32.1) and *TOP1 *(20q12) were found in a second pre-malignant lesion and *DMBT1 *(10q25.3), *ERBB2 *(17q12), and 4qTEL11 (4qtel) amplification was found in the third sample from this group.

In the invasive tumors and the cell lines, the most common amplifications (58.8% of the samples) were found at the clones *RBP1-RBP2 *(retinol binding protein 1 and 2, 3q21-q22), present as a high copy number amplification (HCNA) in two samples; *DAB2 *(disabled homolog 2, mitogen-responsive phosphoprotein (*Drosophila*), 5p13; C84C11/T3 (5ptel) and D5S23 (5p15.2), followed by gains of *Tp63 *(3q27-q29, 2 HCNA); *EGFR *(Epidermal growth factor receptor, 7p12.3-p12.1, 4 HCNA) and D5S2064 (5p15.2), in 52.9% of the invasive samples and amplification of *INS *(Insulin, 11ptel) in 47% of the samples.

**Figure 1:**
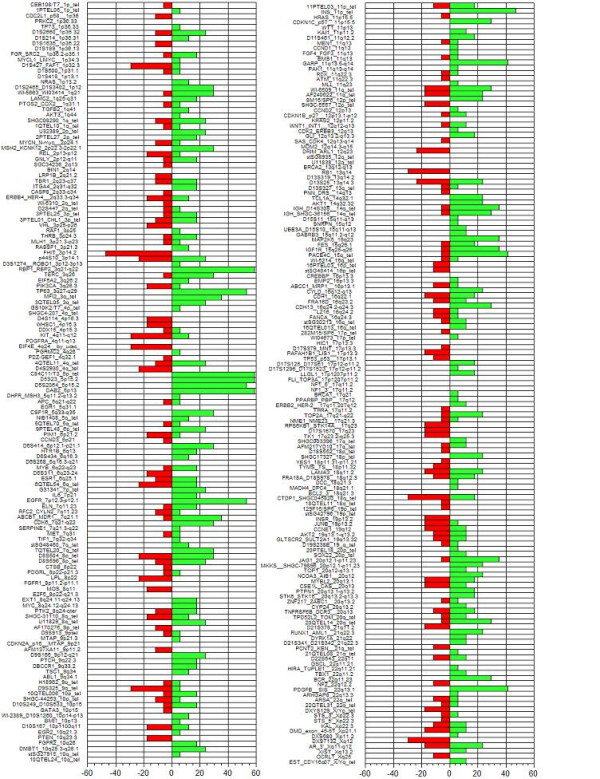
Histogram showing the incidence of alterations in the invasive tumors and cell lines in each of the targets printed on the CGH array. The incidence value is shown at the bottom of the figure; negative values indicate DNA losses, positive values DNA gains.

The most common deletion was found at the clone corresponding to the *FHIT *(Fragile histidine triad) gene (3p14.2), present in 47% of the invasive samples, followed by deletions at D8S504 (8p23.3), *CTDP1-SHGC*- 145820 (18qtel), *KIT *(4q11-q12), D1S427-*FAF1 *(1p32.3), D9S325 (9qtel), *EIF4E *(eukaryotic translation initiation factor 4E, 4q24), *RB1 *(13q14), and DXS7132 (Xq12) present in 29.4% of the samples. A histogram of the DNA copy number alterations detected in the tumor samples analyzed by array CGH is presented in figure 1. The results of these experiments can be accessed through the Progenetix CGH database [25].

## Discussion

Previous studies using chromosomal CGH have delimited a specific pattern of chromosomal imbalances in cervical carcinoma. However, there is little knowledge regarding the identity of particular genes that might be the targets for these copy number changes, making microarray CGH an attractive method in order to define these particular gene targets.

There was concordance between the alterations detected by microarray CGH and the pattern of chromosomal alterations already described by chromosomal CGH. Although the array that we used did not cover the entire genome, we were able to detect alterations at particular genes and genetic markers that might be related to the transformation process in the cervical epithelium.

It is important to notice that the limited number of pre-malignant lesions analyzed did not allowed us to detect any particular region that might be related with this stage of the disease. However, an interesting candidate gene amplified in one pre-malignant sample and in 5 invasive tumors was *MSH2-KCNK12 *(2p22.3-2p22.1). This gene is the human homolog of the *E. coli *mismatch repair gene *mutS*, and has been found mutated in hereditary nonpolyposis colon cancer. Higher *MSH2 *expression has been described in cervical intraepithelial neoplasias and invasive cervical carcinomas than in non-neoplastic cervical lesions. An altered expression of this gene has also been proposed as an important event during cervical carcinogenesis [26,27]. Interestingly, the invasive samples showing *MSH2 *amplification presented with a high number of alterations (>40), suggesting a possible connection between increased copy number of this gene and chromosomal instability in invasive cervical carcinomas.

One of the most important genetic events during cervical carcinoma progression is the gain of 3q. This alteration has been detected in early stages of cervical transformation and in cooperation with other imbalances, seems to play an important role in tumor development. Microarray analysis identified among the most prevalent alterations in cervical tumors and cell lines the amplification of the *RBP1- RBP2 *(Retinol binding protein 1 and 2, 58.8%) and *Tp63 *(52.9% of the samples) genes, located at 3q21-q22 and 3q27-q29, respectively.

*Tp63 *is a homolog of the *p53 *tumor suppressor gene. Its protein is inactivated by the E6 HPV oncoprotein and plays a primordial role in the development of squamo-stratified epithelia. *Tp63 *is highly expressed in the basal stratum of these epithelia with diminished expression in the differentiated strata, suggesting that the presence of this protein preserves the self-renewal capacity of the epithelial stem cells after an asymmetric division, in which one of the daughter cell must conserve its epithelial stem-cell properties and the other daughter cell is committed to the differentiation process [28]. This protein has been detected in human cervical tissues in the basal and parabasal layers of the ectocervical squamous epithelium, and it is not present in the differentiated layers. In premalignant lesions and invasive squamous tumors, a strong *p63 *expression has been described [29,30]. The presence of this protein has also been associated with poor survival and locoregional failure after radiation and chemotherapy [31]. Expression of the epidermal growth factor receptor (*EGFR*, 7p12.3-p12.1), which was found amplified in 52.9% of the invasive tumors that we analyzed, was found to be a prognostic predictor of extrapelvic failure after treatment, and the expression of both molecules was found to be a very good risk factor measurement in patients with stage IIB squamous cell carcinoma of the uterine cervix, who had received radiotherapy and concurrent chemotherapy [31].

*DAB2 *on 5p13 was amplified in 58.8% of the invasive cases. The *DAB2 *gene has been identified as a potent tumor suppressor gene in prostate and ovarian carcinoma [32], and loss of expression of this gene has been associated with the transition of ovarian epithelial cells to premalignant states [33]. *DAB2 *has been implicated in cell positioning control and seems to mediate the requirement for basement membrane attachment of epithelial cells [34]. To our knowledge, there are no available reports analyzing the expression of this gene in the uterine cervix or in cervical carcinoma. The amplification of this gene seems contrary to its putative role as a tumor suppressor gene. A possible explanation for this observation might be the loss of one allele followed by the amplification of the remaining chromosome. However, since array CGH does not offer any type of information regarding the parental origin of the amplified chromosome, this situation can not be confirmed.

Detection of the TERC gene amplification has been recently proposed as a potential marker for the evaluation of cervical carcinoma progression [35]; however, we detected amplification of the clone representing this gene in less than 10% of the samples analyzed by CGH arrays. FISH analysis, as described by Heselmayer *et al., *detected a higher prevalence of nuclei with a diploid pattern than those with a tetraploid pattern, even in the high grade lesions. Furthermore, the percentage of nuclei with more than 2 copies of 3q, including the tetraploid cells ranged between 3.3 to 50% of the CIN3 (cervical intraepithelial neoplasia grade 3). Dellas et al., [9] used in situ hybridization to analyze the prevalence of 3q amplifications in cervical cancer tissue arrays, detecting low level amplifications in most of the tumors studied. These results suggest that these low copy number gains might not be adequately detected by chromosome or even array CGH, due to the contamination with normal cells and/or the presence of a high number of diploid or tetraploid cells in the sample.

Regarding DNA losses, the *FHIT *(fragile histidine triad, 3p14.2) gene suffered losses in 47% of the cases. Aberrant expression of this gene has been well documented in cervical carcinoma and has been related to lymph node metastasis, parametrial invasion, and vaginal involvement in invasive tumors [36]. An association between *FHIT *gene abnormalities and infection with particular HPV types has been suggested, since 87% of the cases with absent *FHIT *expression were positive for HPV16 infection [37]. Furthermore, abnormal expression of this gene has been found in significantly younger patients than those with normal expression, suggesting that abnormalities in the regulation of this gene might be accelerating carcinogenesis in cooperation with HPV [37]. These observations might be related to the preferential integration of HPV into fragile sites, particularly FRA3B, where *FHIT *is located [38].

## Conclusion

In conclusion, microarray CGH allowed the detection of particular genes located in regions with common DNA copy number changes in cervical carcinoma. Further studies using CGH arrays with a higher resolution and the possibility to combine LOH with copy number changes, might be useful for the detection of gene specific targets that are relevant for the genesis and progression of cervical carcinoma.

## Abbreviations

CGH: Comparative Genomic Hybridization, UCC: Uterine cervix carcinoma, HPV: Human papilloma virus, DAPI: 4,6-diamino-2-phenylindole.

## Competing interests

The author(s) declare that they do not have any competing interests.

## Authors' contributions

AH: Performed the microarray CGH experiments, data analysis and paper writing; MB: Help with data submission to the Progenetix database, data analysis; IP: provided training for the experiments, CGH data analysis; PP: tissue processing; GV: HPV typing; DH: sample collection; JG: provided access to the samples; ML: access to samples, histopathological analysis; RL: sample collection; CP: DNA extraction; JG: Help with data analysis; KV: DNA extraction; BA: HPV typing; MS: project coordinator.

## Pre-publication history

The pre-publication history for this paper can be accessed here:



## Supplementary Material

Additional File 1 Raw data from the CGH microarray experiments. This is text file containing the raw data from the microarrays, the first column denotes the identification of the clone, column 2 represents the cytogenetic position of the clone, sample data begins in column 3. Click here for file

## Figures and Tables

**Table 1:** Clinical stage and HPV status of the analyzed samples.

**Sample**	**Stage**	**HPV**
*Pre-malignant*		
G60	CIN I	58
G67	CIN III	16
G42	CIN III	31
*Invasive tumors*		
T56	Ib	16
T55	Ib	16
T46	Ib	16
T24	IIb	Undetected
SXA	IIb	16
LRL	IIb	16
ASJ	IIb	16
A69	IIIb	Undetected
VGR	IIb	Undetected
T49	IVa	16
*Cell lines*		
CaLo	IIb	18
ViBo	IIb	18
INBL	IVa	18
RoVa	IVa	18
HeLa		18
SiHa		16
CasKi		16
